# Statins’ Effect on Cognitive Outcome After Traumatic Brain Injury: A Systematic Review

**DOI:** 10.7759/cureus.16953

**Published:** 2021-08-06

**Authors:** Waleed Sultan, Alisha Sapkota, Hajra Khurshid, Israa A Qureshi, Nasrin Jahan, Terry R Went, Jerry Lorren Dominic, Myat Win, Amudhan Kannan, Anjli Tara, Sheila W Ruo, Michael Alfonso

**Affiliations:** 1 Medicine, California Institute of Behavioral Neurosciences & Psychology (CIBNP), Fairfield, USA; 2 Psychiatry, California Institute of Behavioral Neurosciences & Psychology (CIBNP), Fairfield, USA; 3 General Surgery, California Institute of Behavioral Neurosciences & Psychology (CIBNP), Fairfield, USA

**Keywords:** statins, traumatic brain injury, dementia, alzheimer's disease, concussion, cognitive decline

## Abstract

Traumatic brain injury (TBI), also known as the "Silent Epidemic," is a growing devastating global health problem estimated to affect millions of individuals yearly worldwide with little public recognition, leading to many individuals living with a TBI-related disability. TBI has been associated with up to five times increase in the risk of dementia among multiple neurologic complications compared with the general population. Several therapies, including statins, have been tried and showed promising benefits for TBI patients. In this systematic review, we evaluated the recent literature that tested the role of statins on neurological and cognitive outcomes such as Alzheimer’s Disease and non-Alzheimer’s dementia in survivors of TBI with various severities.

We conducted a systematic search on PubMed, PubMed Central, MEDLINE, and Google Scholar. MeSH terms and keywords were used to search for full-text randomized clinical trials (RCTs), cross-sectional, case-control, cohort studies, systematic reviews, and animal studies published in English. Inclusion and exclusion criteria were applied, and the articles were subjected to quality appraisal by two reviewers.

Our data search retrieved 4948 nonduplicate records. A total of 18 studies were included - nine human studies, and nine animal laboratory trials - after meeting inclusion, eligibility, and quality assessment criteria. Simvastatin was the most tested statin, and the oral route of administration was the most used. Eight human studies showed a significant neuroprotective effect and improvement in the cognitive outcomes, including dementia. Four randomized clinical trials with 296 patients showed that statins play a neuroprotective role and improve cognitive outcomes through different mechanisms, especially their anti-inflammatory effect; they were shown to lower tumor necrosis factor (TNF)-α and C-reactive protein (CRP) levels. Also, they decreased axonal injury and cortical thickness changes. In addition, four cohort studies compared a total of 867.953 patients. One study showed a decrease in mortality in statin-treated patients (p=0.05). Another study showed a reduction in the incidence of Alzheimer's disease and related dementias (RR, 0.77; 95% CI, 0.73-0.81), while one study showed a decreased risk of dementia after concussions by 6.13% (p=0.001). On the other hand, one cohort study showed no significant difference with the use of statins.

In eight animal trials, statins showed a significant neuroprotective effect, improved cognitive outcomes, and neurological functions. Different molecular and cellular mechanisms were suggested, including anti-inflammatory effects, promoting angiogenesis, neurogenesis, increasing cerebral blood flow, neurite outgrowth, promoting the proliferation and differentiation of neural stem cells, and reducing axonal injury. On the contrary, one study showed no benefit and actual adverse effect on the cognitive outcome.

Most of the studies showed promising neuroprotective effects of statins in TBI patients. Cognitive outcomes, especially dementia, were improved. However, the optimal therapeutic protocol is still unknown. Thus, statins are candidates for more advanced studies to test their efficacy in preventing cognitive decline in patients with TBI.

## Introduction and background

Traumatic brain injury (TBI), which is known as the “silent epidemic” [1], continues to be a growing devastating public health problem with little public recognition. TBI represents the greatest contributor to death and disability globally among all trauma-related injuries with significant medical, familial, social, and economic consequences [2,3]. It is estimated that around sixty-nine million individuals suffer TBI from all causes of injury each year worldwide [4]. Data from the CDC indicate that each year in the USA, 2.8 million people sustain a TBI with approximately 56.000 TBI-related annual deaths [5], and around 5.3 million people are living with a TBI-related disability [6, 7]. TBI commonly leads to neurologic complications such as seizures, impaired attention, Alzheimer’s disease (AD), or poor executive function, and mental health issues [8]; for example, 30-70% of TBI survivors can develop depression [7]. Besides, it has long been recognized that severe TBI in early and mid-life is associated with five times increased risk of late-life dementia compared with the general population [9, 10]. TBI survivors also exhibit increased poor decision-making and impulsive-aggressive behavior. Such impairments can affect interpersonal relationships, contribute to poor social integration, and lead to long-term institutional therapy [7]. Literature has shown that only about 25% of people may achieve long-term functional independence following TBI [8].

**Figure 1:**
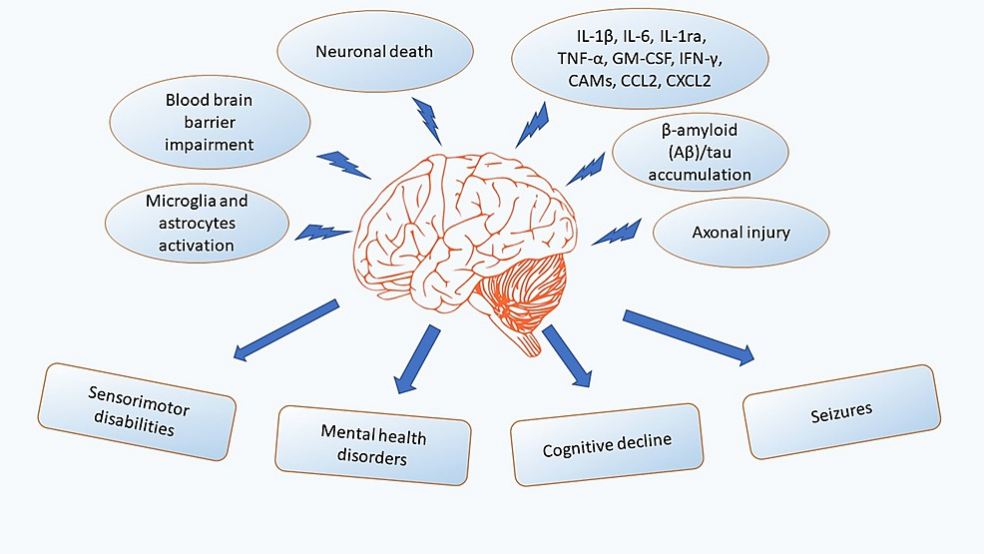
The chronic neuroinflammatory response to TBI in the brain and the complications connected to it. TNF-α: Tumor necrosis factor-alpha, IL: interleukin, GM-CSF: Granulocyte-macrophage colony-stimulating factor, CCL2: C-C motif chemokine ligand 2, CXCL2: C-X-C motif chemokine ligand 2, IFN-γ: Interferon-gamma, CAMs: Cell adhesion molecules.

Recent studies have linked TBI etiologically to Alzheimer's disease (AD) and non-Alzheimer’s dementia (non-AD). β-amyloid (Aβ) and tau proteins deposition after TBI is associated with the development of post-concussive syndrome, dementia, and chronic traumatic encephalopathy (CTE) [11]. Epidemiological studies show that 30% of patients who die of TBI have Aβ plaques, which are pathological features of AD; thus, TBI acts as an important epigenetic risk factor for AD [12, 13, 14]. Various evidence in human and animal studies support that prolonged neuroinflammation is linked to neurodegeneration and the onset of chronic symptoms in TBI [13]. Chronic neuroinflammation is supported by evidence of existing microglia activation lasting for decades after TBI [13, 15]. In addition, recent evidence suggests that TBI causes diffuse axonal injury (DAI) and may induce long-term neurodegenerative processes, such as insidiously progressive axonal pathology [15]. Multiple studies demonstrated that the apoptotic mechanism contributes to the overall pathology of TBI; current studies have suggested the association of neuronal damage through an apoptotic pathway even with mild TBI [16]. Additionally, in TBI, nerve cells release enhanced extracellular glutamate that provokes neurotoxicity; it overstimulates glutamate receptors leading to excessive calcium entry, causing excitotoxicity and neuronal death [17]. The neuroinflammatory response to TBI and complications connected to it are summarized in Figure 1.

While there is no specific pharmacological agent that will definitely improve the clinical outcomes after TBI, several therapies have shown promising benefits for TBI patients. Especially 3-Hydroxy-3-methylglutaryl coenzyme A (HMG CO-A) reductase inhibitors, also known as statins, are recognized for their cholesterol-lowering efficacy. They are also attracting significant interest in treating and preventing neurodegenerative diseases [18]. The neuroprotective mechanisms thought to underlie statins’ role in the prevention of cognitive impairment are varied. Statins’ role in the reduction of neuroinflammation is a common theory. Rosuvastatin showed a significant effect on the reduction of tumor necrosis factor-alpha (TNF-α) levels in a randomized clinical trial [19], and simvastatin alleviates toll-like receptor 4 (TLR4)-mediated inflammatory injury; at the same time, it promotes neurological recovery and resists oxidative stress through the nuclear erythroid 2-related factor 2- antioxidant response element (Nrf2-ARE) signaling pathway, thus exerting a neuroprotective effect in TBI. Also, the current research in rat models demonstrates that statins reduce axonal injury significantly and improve functional outcomes after experimental TBI [20]. Moreover, lowering cholesterol may be used in neuroprotection in cases with excessive extracellular glutamate. TBI increases in the reversal of glutamate uptake. A decrease in the level of membrane cholesterol will attenuate the transporter-mediated glutamate release from nerve terminals and may contribute to the neuroprotective effects of statins [17].

The effects of statins on cognitive function, especially following TBI, have received increasing attention in recent years. However, data from different studies have been inconsistent, not only in results but also in analytical methods, population characteristics, pre-existent cognitive impairments, statins studied, and the endpoints used [21]. There is also a deficiency in information on TBI-related AD and non-AD dementia pathophysiology, including the pharmacokinetics and pharmacodynamics of statins in the brain [18]. This systematic review aims to discuss whether statins can be beneficial in improving the cognitive outcomes in survivors of TBI of various severities.

## Review

Methods

Search Strategy

**Figure 2:**
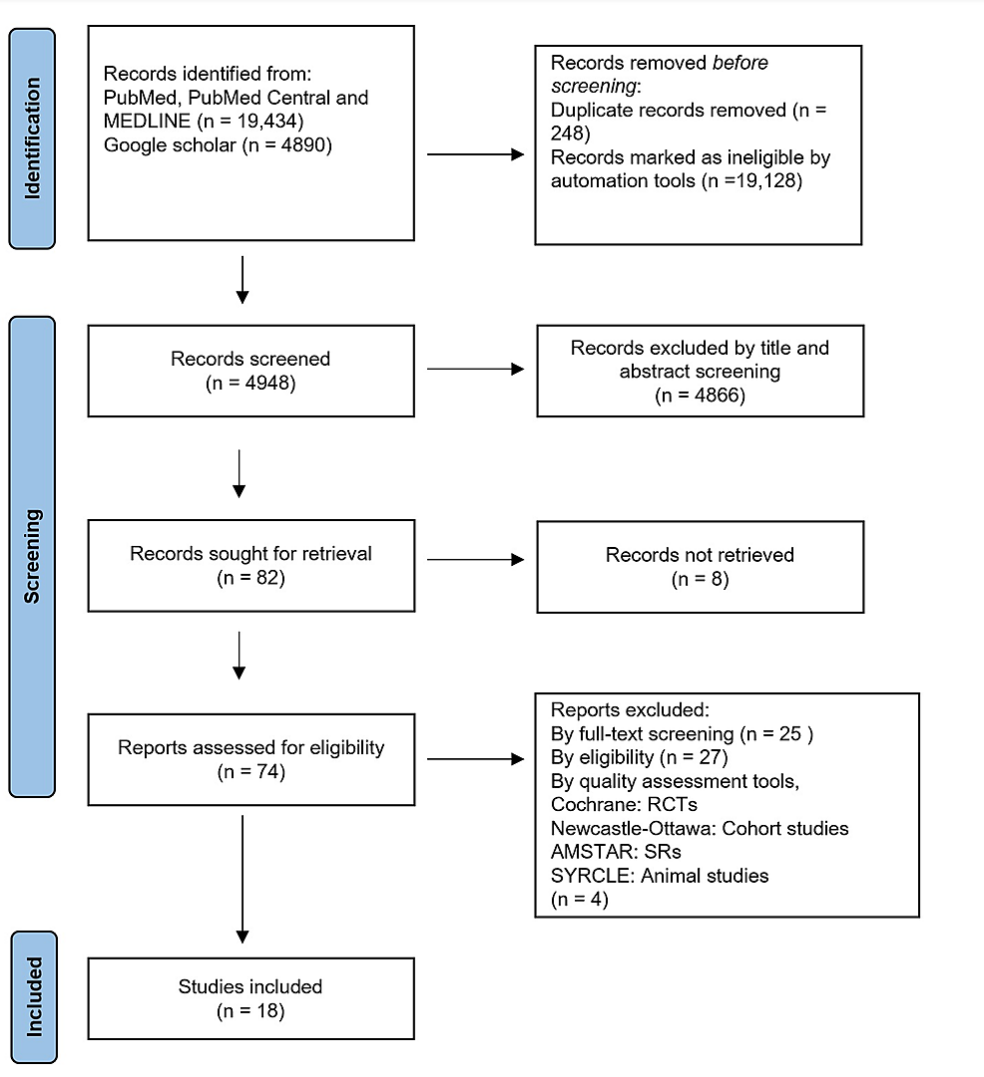
PRISMA flow diagram 2020 showing the search results and selection process. PRISMA: Preferred Reporting Items for Systematic Review and Meta-Analyses, RCT: Randomized clinical trial, AMSTAR: Assessment of multiple systematic reviews, SRs: Systematic reviews, SYRCLE: Systematic review centre for laboratory animal experimentation

We followed the Preferred Reporting Items for Systematic reviews and Meta-Analyses (PRISMA) guidelines in our systematic review. As shown in Figure 2, we conducted a systematic search on PubMed, PubMed Central (PMC), Medical Literature Analysis and Retrieval System Online (MEDLINE), and Google Scholar on April 27, 2021.

We used terms of medical subject heading (MeSH) and keywords for the topic of interest like “Traumatic brain injury,” “statins,” “cognitive dysfunction,” “Dementia,” “Alzheimer’s” separately and in combination (Table 1), we searched for full-text randomized clinical trials (RCTs), case-control, cross-sectional, cohort studies, systematic reviews, and animal studies published in the English language in the last 10 years i.e. from April 27, 2011, to April 27, 2021.

**Table 1:** Keywords, MeSH terms used, and results.

Keywords	Database	Initial results	After screening	Eligible
“Statins” AND “Cognitive outcome” AND “Traumatic brain injury” & Mesh terms.	PubMed, PubMed Central (PMC), MEDLINE	19,434	52	13
“Statins” AND “Cognitive outcome” AND “Traumatic brain injury”	Google Scholar	4,890	22	5

Inclusion Criteria

Only full-text peer-reviewed clinical trials, observational studies, and animal studies that examined outcomes of using statins after TBI published in the past 10 years in the English language were included. Patients had to be on a statin before the TBI or were given statins within 90 days after the trauma; patients had to be free of significant neurological disability before the TBI.

Exclusion Criteria

We excluded articles in languages other than English, grey literature, case reports, letters to the editor, books, documents, studies published before 2011.

Study Selection

Two reviewers (WS and AS) screened the retrieved articles by titles and abstracts and applied quality assessment using the optimal risk of bias tools. We used the Cochrane bias risk tool for clinical trials, the Newcastle-Ottawa bias scale tool for cohort studies, the assessment of multiple systematic reviews (AMSTAR) tool for systematic reviews, and the systematic review center for laboratory animal experimentation (SYRCLE) tool for animal trials, as shown in Table 2.

**Table 2:** The included studies, risk of bias tools used, and total scores. RCT: Randomized clinical trial, AMSTAR: Assessment of multiple systematic reviews, SYRCLE: Systematic review centre for laboratory animal experimentation.

Author & Year	Study Type	Bias Risk Tool	Score
Sánchez-Aguilar et al. 2013 [19]	RCT	Cochrane	7
Naghibi et al. 2016 [22]	RCT	Cochrane	7
Farzanegan et al. 2017 [23]	RCT	Cochrane	7
Govindarajan et al. 2016 [24]	RCT	Cochrane	7
Khokhar et al. 2018 [25]	Cohort	NEWCASTLE - OTTAWA	8
Redelmeier et al. 2019 [26]	Cohort	NEWCASTLE - OTTAWA	7
Li et al. 2020 [27]	Cohort	NEWCASTLE - OTTAWA	7
Mansi et al. 2020 [28]	Cohort	NEWCASTLE - OTTAWA	8
Gruenbaum et al. 2016 [29]	Systematic review	AMSTAR	8
Wang et al. 2014 [30]	Animal clinical trial	SYRCLE	7
Xu et al. 2017 [31]	Animal clinical trial	SYRCLE	8
Wu et al. 2011 [32]	Animal clinical trial	SYRCLE	8
Wu et al. 2012 [20]	Animal clinical trial	SYRCLE	8
Abrahamson et al. 2013 [33]	Animal clinical trial	SYRCLE	7
Xie et al. 2014 [34]	Animal clinical trial	SYRCLE	8
Darwish et al. 2014 [35]	Animal clinical trial	SYRCLE	8
Mountney et al. 2016 [36]	Animal clinical trial	SYRCLE	7
Mountney et al. 2016 [37]	Animal clinical trial	SYRCLE	7

Results

Our primary data search on PubMed, PMC, and MEDLINE resulted in 4036 articles. We identified and eliminated duplicates and then screened 4023 articles by title and abstract. We then applied eligibility criteria and quality assessment tools on 34 relevant articles; we included 13 in the study. Additionally, we retrieved 15 pertinent nonduplicate articles from Google Scholar after screening by title and abstract; we included five studies in the review after meeting inclusion, eligibility, and quality assessment criteria. Finally, we arrived at 18 studies for the study, as shown above in Table 1 and Table 2.

Simvastatin was the most commonly administered statin. Eight human studies showed a significant neuroprotective effect and improved cognitive outcomes. Four RCTs showed that statins played a neuroprotective role and improved the cognitive outcomes through their anti-inflammatory effect, decreased the axonal injury and cortical thickness changes. In addition, four cohort studies supported the positive impact of statins on the survival and cognitive outcomes, including AD and non-Alzheimer’s dementia after TBI. On the other hand, one cohort study showed no significant difference with the use of statins. Eight animal studies showed a significant neuroprotective effect and improved neurological functions. On the contrary, one study showed an adverse effect on the cognitive outcome.

Discussion

Pharmacological Basis

Statins have been long used to treat hyperlipidemia and are used for the primary and secondary prevention of cardiovascular events among older adults with cardiovascular disease (CVD) [18,38]. Currently, the statins approved for use in the US include single-ingredient products such as Lipitor (atorvastatin), Lescol (fluvastatin), Mevacor (lovastatin), Altoprev (lovastatin extended-release), Livalo (pitavastatin), Pravachol (pravastatin), Crestor (rosuvastatin), and Zocor (simvastatin) [39]. Each statin varies in pharmacology. For instance, lovastatin, simvastatin, fluvastatin, and atorvastatin have greater potential to cross the blood-brain barrier (BBB). At the same time, pravastatin, pitavastatin, and rosuvastatin are less likely to enter non-hepatic cells than lipophilic statins [38, 40].

**Figure 3:**
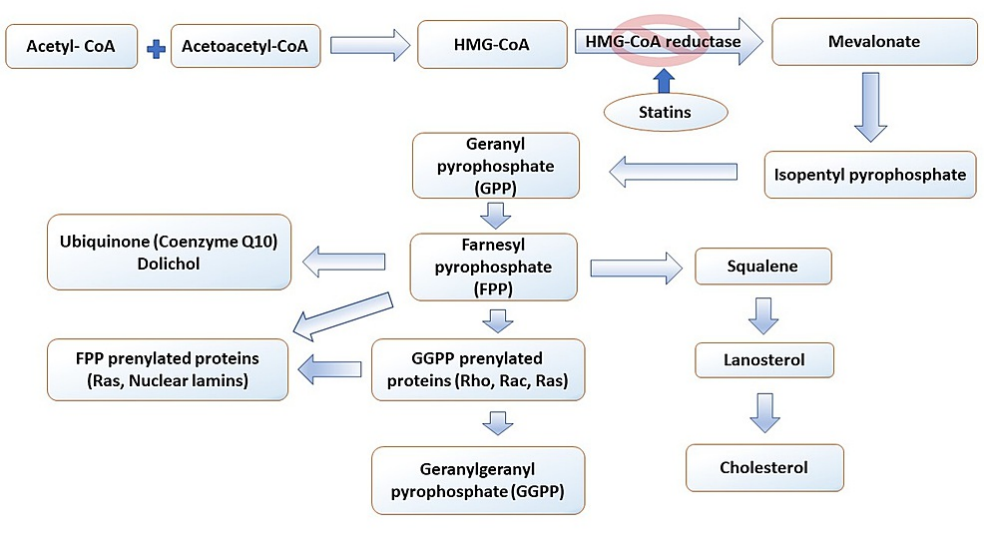
Biosynthesis pathway of mevalonate, isoprenoids, and cholesterol. HMG CO-A:  3-hydroxy-3-methylglutaryl coenzyme A; Statins inhibit HMG-CoA reductase at the rate-limiting step for cholesterol synthesis [18].

Statins' role as neuroprotectors has attracted much attention, but there is a lack of research on the basic pharmacology of statins in the brain. A few studies have shown that statin lactones and acids pass the BBB. However, the active transport mechanisms of these acid forms are not understood [18]. In addition, statins reduce the production of mevalonate, which is the precursor of isoprenoids and cholesterol, as shown in Figure 3. Therefore, the protective effects of statins may be due to reductions in isoprenoids, which decrease prenylated proteins, reduce cholesterol that can alter cell structure and function, lower dolichol and Co-enzyme Q10 (CoQ 10), and effects outside of the mevalonate/isoprenoid/cholesterol pathway such as stimulation of B-cell lymphoma-2 (Bcl-2) gene expression [18,41,42].

Statins’ levels in the brain have a half-life of approximately two to four hours [18,40]. More studies need to determine if statins are metabolized in the brain or actively transported out of the brain as there is little information on the regulation of brain farnesyl pyrophosphate (FPP) and geranylgeranyl pyrophosphate (GGPP), and even less data on the effects of statins on these two isoprenoids. There is also a lack of knowledge of the statins' effects on brain dolichol and CoQ 10. This information would provide insight into specific drug targets and provide a rationale for optimizing statins to cross the BBB and increase their bioavailability in the brain [18].

Pathophysiology and Molecular Basis

Human studies: Statins’ neuroprotective role after TBI arises from their effect on different molecular and cellular mechanisms, including neuroinflammation, apoptosis, brain edema, angiogenesis, and neurogenesis. Some studies support that prolonged neuroinflammation is linked to neurodegeneration and the onset of chronic symptoms in TBI [10]. Furthermore, the elevation in TNF, interleukin 1 beta (IL-1β), interleukin-6 (IL-6), interleukin-8 (IL-8), and interleukin-10 (IL-10) is associated with poor outcomes [10, 11].

An RCT by Sánchez-Aguilar et al. studied the anti-inflammatory effect of statins [19]; they included 36 patients aged 16 to 60 years who presented within 24 hours moderate to severe TBI that caused intracranial lesions confirmed by radiology scanning. Rosuvastatin 20mg was given to 19 patients orally, while 17 patients received a placebo for 10 days. The study showed a significant effect of rosuvastatin on the reduction of TNF-α levels (p=0.004). On the other hand, rosuvastatin treatment did not appear to significantly affect the levels of IL-1β (p>0.05), IL-6 (p=0.091), and IL-10 (p=0.134). The functional outcome was measured using the disability rating scale (DRS) and Galveston orientation amnesia test (GOAT) scores. The rosuvastatin group also showed a significant reduction in the disability scores (p=0.03), indicating a better functional outcome. This study indicates that the role of statins as anti-inflammatory agents that improve the functional outcome needs more attention and research. However, new evidence strongly suggests that the biologically relevant compartment for studying cytokines in TBI should be the extracellular brain [43]. Also, it is worth mentioning that this study included a small number of patients, and they measured disability scores only at three and six months [19].

Naghibi et al. included 43 patients who were 18 years and older with head trauma that required ICU admission and no other types of trauma in a prospective, double-blind RCT [22]; 22 received simvastatin 20mg tablets at a dose of 80mg on the first day and then 40mg daily while 21 received a placebo (lactose). The C-reactive protein (CRP) concentration 48 hours after trauma in the statin group was significantly lower than in the placebo group (p=0.042). The simvastatin group showed a lower IL-6 level 48 hours after trauma than the placebo group. However, this was not statistically significant (p=0.55). The overall ICU mortality rate, the duration of mechanical ventilation, and the length of ICU stay were similar between the groups. The Glasgow coma scale (GCS) score at discharge was higher than at the time of admission in both groups. However, it was significantly higher in the simvastatin group (p=0.004) when compared with the placebo group. These results suggest the anti-inflammatory effect of statins on the cognitive outcome and secondary injuries after TBI. The main limitations of this study were the small sample size and the lack of generalizability as the study was on patients of the same race in one institution.

Farzanegan et al. tested the effect of statins on brain contusion volume and the neurological outcome after TBI in an RCT [23]. The researchers included 64 patients with moderate and severe TBI. The treatment group received atorvastatin 20mg tablets 10 hours after injury, and daily for 10 days (n=32), while the control group received the same dosage placebo (n=32). Non-contrast spiral thin-cut brain CT scans were obtained on admission on the third and seventh day after TBI. The neurological outcome was measured and recorded by a resident blinded to the study groups, using Glasgow outcome scale (GOS), modified Rankin scale (MRS), and DRS at a three-month follow-up. There was no significant difference between the two study groups regarding the baseline, the third day and the seventh day of the contusion volume, nor the rate of expansion of contusions. However, functional outcome scales of the GOS (p=0,043), MRS (p=0.039), and DRS (p=0.030) were significantly better in the atorvastatin group compared to placebo. This study concluded that atorvastatin has no beneficial effect on the expansion rate of cerebral contusions in patients with moderate and severe TBI. However, there were improved functional outcomes in patients with cerebral contusions. The study faced some limitations - the most important one being that many patients were lost to follow-up in both study groups, resulting in a reduced sample size less than targeted. However, that didn't affect the statistical power of the study.

Cortical atrophy in mild TBI (mTBI) has attracted attention recently as underlying pathology for cognitive decline; axonal injury can be the primary cause of cortical thinning [44]. Axonal degeneration is known to continue for years after injury and appears to play a role in developing Alzheimer's disease-like pathological changes [15]. A study by Govindarajan et al. performed a cross-sectional and longitudinal analysis of cortical thickness changes on 135 patients who were either mTBI (n=71) patients or orthopedic trauma controls without TBI (n=59) [24]. Cortical thickness was compared between the mTBI group and the orthopedic control groups by MRI at 24 hours and three months after injury. Cortical thinning occurred in the middle temporal cortex in the left hemisphere and the supramarginal cortex in the right hemisphere at 24 hours post-injury (acute phase point) and the left middle temporal cortex at three months post-injury (recovery phase point) in the mTBI group in comparison to the orthopedic controls (p=0.01). Within the mTBI group, in an RCT of atorvastatin (n=33) versus non-treatment group (n=38), at three months, the treatment group did not show any significant differences compared with the orthopedic control group. Significant cortical thinning was observed in the non-treatment group in the left middle temporal cortex. The main limitation of this study is that the sample size was small. Secondly, a more powerful longitudinal analysis could have been made if the study had more time points throughout the recovery. Thirdly, three months is a short time to observe apparent cortical thinning, and more data needs to be collected further down the recovery phase. In Table 3, we summarize the selected human studies showing the pathophysiological outcomes in our research.

**Table 3:** Summary of the selected human studies showing the pathophysiological effects of statins after TBI RCT: Randomized controlled trial; TNF-α: Tumor necrosis factor α; TBI: traumatic brain injury; IL: Interleukin; GCS: Glasgow coma scale; GOS = Glasgow outcome scale; MRS = Modified Rankin scale; DRS = Disability rating scale; mTBI = Mild traumatic brain injury.

Study author & year	Study type & sample size	Purpose	Intervention	Outcome & Conclusion
Sánchez-Aguilar et al. 2013 [19]	RCT, 36	Effect of rosuvastatin on inflammatory cytokines and functional outcomes after TBI in humans	n=19 rosuvastatin 20mg orally, n=17 placebo, day one - 10	Rosuvastatin ↓↓ TNF-α (p=0.004), -No effect on IL-1β (p>0.05), IL-6 (p=0.091), IL-10 (p=0.134), ↓↓ disability (p>0.03) Rosuvastatin has an anti-inflammatory effect and improves the outcomes after TBI
Naghibi et al. 2016 [22]	RCT, 43	Effect of simvastatin on the outcome of TBI	n=22 oral simvastatin 80mg first day then 40mg daily, n = 21 placebo (lactose)	Simvastatin ↓↓ CRP levels (p=0.042), ↑↑ GCS at discharge (p=0.004) Simvastatin plays an anti-inflammatory role and improves the neurological outcomes after TBI
Farzanegan et al. 2017 [23]	RCT, 64	Effect of atorvastatin on brain contusion volume and functional outcome after moderate and severe TBI	n=32 atorvastatin 20mg oral tablets, daily /first 10 days, n=32 placebo	No significant difference in contusion volume day three (p=0,073), day seven (p=0,082), improved functional outcome GOS (p=0,043), MRS (p=0.039), DRS (p=0.030) Atorvastatin has no beneficial effect on cerebral contusion. However, it was associated with improved functional outcomes.
Govindarajan et al. 2016 [24]	RCT, 135	Cortical thickness changes after mTBI and the effect of statins	n=59 ortho, n=71 mTBI, n=33 atorvastatin, n=38 non-treatment	↑↑ Cortical thinning, and axonal injury (p=0.01) in the non-treatment group Atorvastatin decreases the cortical thickness changes after TBI

Animal studies: Multiple animal studies investigated different mechanisms and effects through which statins could be playing their neuroprotective role. The anti-inflammatory effect drew great attention as in human studies. Wang et al. induced TBI and cortical contusions in adult male Sprague-Dawley rats (n=45; weight=400-450g) by controlled cortical impact (CCI) device [30]. They included three groups: Sham surgery group received craniotomy and distilled water placebo (n=5), control group with TBI and distilled water placebo (n=20), and a treatment group (n=20) with TBI and simvastatin (15 mg/kg) in distilled water daily for three days via an orogastric tube inserted each day, and the first dose was given one hour after TBI. They used a grip test (Grip strength meter, Singa) to assess the neurological function before TBI, at 24 hours, 72 hours, and one week after. They used Western blot to measure the levels of intercellular adhesion molecule-1 (ICAM-1) also known as cluster of differentiation 54 (CD54), as a marker of endothelial activation and inflammation. And the reason why ICAM-1 is a marker of inflammation is that it interacts with leukocyte integrins forming firm adhesion of these activated neutrophils through diapedesis across the endothelium [45]. Then, the treatment group had a significantly smaller amount of reduction in successful trials of gripping than the control group (p<0.05). Additionally, the control group had a significant increase in ICAM-1 expression post-injury at the 72-hour time point as compared to that of the simvastatin treatment group (p<0.05) [30]. The authors concluded that the statin treatment was associated with attenuation in the vascular endothelial inflammatory response and decrease in the neurological dysfunction after TBI, ICAM-1 levels were highest at 24 to 72 hours after TBI, which suggests that ant-inflammatory treatment may be most beneficial at 24 to 72 hours after TBI.

Xu et al. conducted another laboratory trial [31] where they induced TBI by CCI device after performing a craniotomy in male C57 black 6 (C57BL/6) mice, while they performed a craniotomy only on the sham group. They randomly assigned the 150 mice to four groups: (1) sham plus saline group (n = 30); (2) sham plus 1mg/kg/day atorvastatin group (n = 30); (3) TBI plus saline group (n = 45); (4) TBI plus 1mg/kg/day atorvastatin group (n = 45). They performed a dose-response analysis using three different doses (1, 5, and 10mg/kg/day). Researchers evaluated neurological outcomes using the modified neurological severity score (mNSS) and rotarod apparatus that assesses fine motor coordination and learning. Enzyme-linked immunoassay (ELISA) was used to measure pro and anti-inflammatory mediators. Atorvastatin (one, five, and 10mg/kg/day) significantly lowered mNSS scores compared with the saline group at 72 hours (p<0.01, 0.05, and 0.01, respectively). However, there was no significant difference among different dose groups. In the rotarod test, the sham groups exhibited the best performance (p<0.001) - mice treated with atorvastatin showed early improvement on the rotarod test, starting at 24 hours post-TBI, compared to the saline group (p<0.05). But there were no significant differences among the three atorvastatin groups(p>0.05). Atorvastatin reduced T cells (CD45+, CD3+) counts significantly in the brain at 72 hours (p<0.001), whereas there were no changes in the invasion of B cells (CD45+, B220+) (p>0.05). Moreover, they detected significantly fewer neutrophils (CD11b+CD45highLy6G+) and natural killer (NK) cells (CD45, NK1.1) in brain samples of the atorvastatin group at 24 and 72 hours. Atorvastatin increased regulatory T lymphocytes (T-regs) in the spleen and the injured brain. It also increased the levels of the anti-inflammatory cytokines Transforming growth factor beta 1 (TGF-β1) at 24 hours (p<0.05) and 72 hours (p<0.001) and IL-10 at 24 hours (p<0.05) and 72 hours (p<0.01) compared with those in the saline group. Meanwhile, the levels of the pro-inflammatory cytokines interferon gamma (IFN-γ) at 24 and 72 hours (p<0.001) and IL-6 at 24 and 72 hours (p<0.001). Moreover, atorvastatin reduced the chemokines regulated on activation, normal T cell expressed and secreted (RANTES) at 24 hours (p< 0.001) and 72 hours (p<0.05), and IFNγ inducible protein (IP-10) at 24 hours (p<0.05) and 72 hours (p<0.001). The results suggested that atorvastatin improved the neurological outcome by attenuating the invasions of T cells, neutrophils, and NK cells, the production of pro-inflammatory cytokines (IFN-γ and IL-6), and chemokines (RANTES and IP-10).

Promoting angiogenesis is another important mechanism that Wu et al. tested in a lab trial [32]. They randomly divided 36 male Wistar rats into three groups (n=12) each. Modified CCI was used to induce TBI. The first group were subjected to TBI and treated with saline placebo, the second group were subjected to TBI and were treated with simvastatin (1mg/kg/day) orally one day later, and for the 14 following days while the third group was treated with sham surgery and saline placebo. They sacrificed four rats in each group on day three. Cortical tissues in the lesion boundary zone were harvested for ELISA of vascular endothelial growth factor receptor-2 (VEGFR-2); they sacrificed eight other rats on day 14. The foot-fault test was performed before TBI and on the first, third, seventh, and 14th day by a blinded investigator to measure the neurological function. Simvastatin reduced the incidence of foot-faults significantly from four days (p= 0.003) and persisted till day 14 (p=0.01) compared to saline, though, there was no significant difference between the simvastatin and the sham groups on day 14 (p=0.055). They measured vascular perimeter with von Willebrand factor (vWF) staining that showed a significant increase in the simvastatin group compared with the control group (p=0.008) in the cortex and (p= 0.006) in the hippocampus. Additionally, simvastatin increased the bromodeoxyuridine (BrdU)-positive endothelial cells (p=0.001), indicating that simvastatin promoted the proliferation of endothelial cells after TBI. Also, simvastatin induced the phosphorylation of endothelial nitric oxide synthase (eNOS) in the injured cortex (p=0.002), and the phosphorylation of protein kinase B (PKB/Akt) and glycogen synthase kinase 3 (GSK-3) increased in the simvastatin group. Moreover, simvastatin increased the expression of VEGFR-2 (1634.1 ± 151 pg/mg) (p=0.02). The authors concluded that simvastatin significantly improved neurological functions through induction of angiogenesis after TBI.

Wu et al. investigated the role of statins in attenuating axonal injury and neurite outgrowth in neurological recovery after TBI [20]. They included 36 male Wistar rats and divided them into three groups of 12 each. The first group had sham surgery; the second group had TBI by modified CCI and saline treatment; the third group also had TBI and oral simvastatin 1mg/kg/day for 14 days. They measured neurological functions by mNSS, amyloid-β precursor protein (APP), and traumatic axonal injury by immunohistochemical staining [46]. TUJ-1(β-tubulin III) immunostaining was performed to identify neurons and neurite outgrowth, and neurons were harvested after 24 hours and analyzed by Western blot and ELISA. Simvastatin significantly reduced the mNSS at day seven (p=0.028) and day 14 (p=0.012) compared to saline, and the score was inversely correlated with the axonal density of the hemisphere ipsilateral to the injury (p=0.008). Simvastatin significantly decreased the density of APP positive axons in the ipsilateral hemisphere (p=0.021). In addition, axons were identified by fluorescent immunostaining of a pan-axonal high molecular weight neurofilament protein (NF-H) marker, SMI-312, and Bielschowsky silver staining. Simvastatin treatment significantly increased the density of NF-H-positive axons in the lesion boundary zone (p=0.007) and density of Bielschowsky silver-positive axons in the striatal bundles (p=0.015). Moreover, simvastatin increased the density of synaptophysin in the ipsilateral hemisphere (p=0.037), simvastatin suppressed the expression of APP while increasing the levels of NF-H and synaptophysin 14 days after injury. These data confirmed that simvastatin reduced axonal injury and increased axonal and synaptic density post-TBI [20]. Simvastatin also significantly increased neurite outgrowth compared with vehicle control in the injured plasma cell neoplasms (PCNs) (p=0.015). Neurite outgrowth was not altered with a cholesterol supplement, suggesting that the effect of simvastatin was independent of cholesterol change. Simvastatin-induced neurite outgrowth was abolished by phosphatidylinositol 3-kinase inhibitor (LY294002) (p=0.022) and enhanced by lithium chloride (LiCl) (p=0.006). Moreover, simvastatin promoted the phosphorylation of Akt, mammalian target of rapamycin (mTOR), and glycogen synthase kinase 3 (GSK-3β) in the injured neurons and increased dephosphorylated activated protein C (APC) levels in the injured neurons. This study demonstrates that simvastatin reduces axonal injury, promotes neurite outgrowth, and improves functional outcomes after TBI [20].

Studies showed that the alterations in brain concentrations of amyloid beta (Aβ) peptide influence cerebral blood flow (CBF). Both increases and decreases in CBF are associated with poor neurologic outcomes after TBI [47] and increase the risk of developing AD [48]. Abrahamson et al. performed a lab study to examine if CBF changes are influenced by human Aβ and are responsive to statins [33]. Male APPNLh/NLh (n=43) and wild-type C57Bl/6J (n=23) mice were divided into different groups: (1) naive (wild-type C57); (2) naive (APPNLh/NLh); (3) CCI, three-day survival (wild-type C57); (4) CCI, three-day survival (APPNLh/NLh), (5) CCI, three-week survival (wild-type C57); (6) CCI, three-week survival (APPNLh/NLh). Simvastatin (3mg/kg) was introduced to APPNLh/NLh mice only. They used arterial spin-labeling magnetic resonance imaging to examine changes in CBF three days and three weeks after CCI injury. The APPNLh/NLh group showed sustained elevations in human Aβ peptides bilaterally in the hippocampus and cortex, which were suppressed by simvastatin treatment except for the ipsilateral hippocampus. Simvastatin significantly increased the CBF in the contralateral cortical contusion region and concomitantly reduced the Aβ levels (p<0.05). The outcomes of this study support the therapeutic potential of simvastatin in human neurological disorders. Limitations to this study included the close correlation between Aβ oligomers and CBF changes that may be masked by the ELISA protocol, which does not distinguish between Aβ monomers and oligomers.

The Notch signaling pathway has a critical role in multiple cell functions such as differentiation, proliferation, and apoptosis. Simvastatin was shown to regulate this pathway [49, 34]. Xie et al. investigated the simvastatin’s effect on proliferation and differentiation of the neural stem cells (NSCs) after TBI and the Notch-1 signaling role in the process [34]. They divided male Wistar rats into three groups (n= 28/group): (1) sham surgery the only group, (2) the control group received CCI-induced TBI plus saline treatment orally, (3) simvastatin group, CCI induced TBI plus 1mg/kg simvastatin was mixed with water and administered directly into the esophagus using a lavage tube starting at day one. Rats were sacriﬁced at one, three, seven, 14, 21, 28, 35 days after TBI (n=4 for each time point), and brain sections were obtained and processed. Neurological functions were measured using mNSS at days one, three, seven, 14, 21, 28, and 35. Flow cytometry was used to evaluate the differentiation of NSCs, astrocytes, and neurons immunostaining for glial fibrillary acidic protein (GFAP) and microtubule-associated protein-2 (MAP-2), respectively. Both MAP-2-positive and GFAP-positive cells were observed after simvastatin administration, suggesting that it induced NSCs differentiation into both (p<0.05). Also, simvastatin signiﬁcantly decreased the mNSS at day 35 (p<0.05); this suggests that chronic simvastatin administration improved neurological functions after TBI. Table 4 summarizes the animal studies that have investigated the effects of statins related to their neuroprotective role.

**Table 4:** Summary of the selected animal trials showing the effect of statins on neuroinflammation, cerebral blood flow, angiogenesis, and neurogenesis after TBI. CCI: Controlled cortical impact; ICAM-1: Intercellular adhesion molecule-1; TBI: Traumatic brain injury; mNSS: Modified neurological severity score; IFN-γ: Interferon-gamma; RANTES: Regulated upon activation normal T-cell expressed and secreted; IL-6: Interleukin-6; Aβ: Amyloid beta peptide; NK cells: Natural killer cells; IP-10: Interferon-γ inducible protein; APP: Amyloid-β precursor protein; TGF-β1: Transforming growth factor-beta1; VEGFR-2: Vascular endothelial growth factor receptor-2; NSCs: Neural stem cells; BrdU: Bromodeoxyuridine; eNOS: Endothelial nitric oxide synthase; CBF: Cerebral blood flow; GFAP: Glial fibrillary acidic protein

Study author & year	Type & characteristics	Intervention	Outcome & Conclusion
Wang et al. 2014 [30]	Animal trial, male Sprague-Dawley rats (n=45)	Sham craniotomy (n=5); control CCI + no treatment (n=20); treatment group (n=20) CCI + simvastatin (15mg/kg) orogastric tube	Simvastatin ↓↓ trials of gripping (p<0.05), control group ↑↑ ICAM-1 at 72 hours (p<0.05) Simvastatin attenuates the vascular endothelial inflammatory response and decreases the neurological dysfunction in TBI
Xu et al. 2017 [31]	Animal trial, C57BL/6 mice (n=150)	Sham+saline group (n=30); sham + 1mg/kg/day atorvastatin (n=30); CCI + saline group (n=45); CCI + 1mg/kg/day atorvastatin group (n=45) ; doses (1, 5, and 10mg/kg/day)	Atorvastatin ↑↑ mNSS scores (p<0.001) to sham, and no dose differences (p>0.05) Atorvastatin (1, 5, 10mg/kg/day) ↓↓ mNSS compared to saline at 72 hours (p<0.01, 0.05, ,0.01) ↓↓ CD45^+ ^, CD3^+^ (p<0.001) & neutrophils (CD11b^+^, CD45^high ^, Ly6G^+^)& NK cells CD45^+^NK1.1^+^, -↑↑ TGF-β1 at 24 h (p<0.05) , 72 hours (p<0.001), IL-10 at 24 hours (p<0.05), 72 h ours (p<0.01) ↓↓ IFN-γ (p<0.001) IL-6 24 (p<0.01) ↓↓ RANTES at 24 hours (p< 0.001) and 72 hours (p< 0.05), IP-10 at 24 hours (p<0.05) and 72 hours (p<0.001) Atorvastatin improved the neurological outcome and attenuated the inflammatory response
Wu et al. 2011 [32]	Animal trial, male Wistar rats (n=36)	CCI + saline (n=12); CCI + simvastatin 1mg/kg/day (n=12); sham (n=12)	Simvastatin ↓↓ incidence of foot-faults at four days (p= 0.003) and 14 days (p=0.01), ↑↑ vascular perimeter (p= 0.008) in cortex and (p= 0.006) in hippocampus, ↑↑ BrdU-positive endothelial cells (p= 0.001), ↑↑ phosphorylation of eNOS (p=0.002), ↑↑VEGFR-2 expression (1634.1 ± 151 pg/mg) (P= 0.02), Simvastatin promotes angiogenesis and improves neurological outcomes after TBI
Wu et al. 2012 [20]	Animal trial, male Wistar rats (n=36)	Sham (n=12); CCI + saline (n=12); CCI + simvastatin 1mg/kg/day (n=12);	Simvastatin ↓↓ mNSS at day seven (p=0.028) & day14 (p=0.012), ↓↓ density of APP positive axons (p=0.021), ↑↑ density of NF-H-positive axons (p=0.007) & Bielschowsky silver-positive axons in the striatal bundles (p=0.015) & synaptophysin (p=0.037), ↑↑ neurite outgrowth (p=0.015), Simvastatin reduces axonal injury, promotes neurite outgrowth, and improves functional outcomes after TBI
Abrahamson et al. 2013 [33]	Animal trial. male APP^NLh/NLh ^(n=43), wild-type C57Bl/6J (n=23) mice	(1) naive (wild-type C57); (2) naive (APP^NLh/NLh^); (3) CCI, three-day survival (wild-type C57); (4) CCI, three-day survival (APP^NLh/NLh^), (5) CCI, three-week survival (wild-type C57); (6) CCI, three-week survival (APP^NLh/NLh^), oral simvastatin (3mg/kg) to APP^NLh/NLh^	Simvastatin ↑↑ CBF in contralateral cortical contusion region & ↓↓ Aβ levels (p<0.05), Simvastatin may decrease the risk of AD via increasing CBF and decreasing Aβ peptide levels after TBI
Xie et al. 2014 [34]	Animal trial, male Wistar rats (n=84)	Sham (n=28), control group received CCI plus saline orally (n=28), CCI + simvastatin 1mg/kg, simvastatin by lavage (n=28)	Simvastatin ↑↑ NSCs differentiation into b (p<0.05) MAP-2-positive and GFAP-positive cells, ↓↓ mNSS at day 35 (p<0.05) Simvastatin enhanced neurological functional recovery after TBI through activation of Notch signaling and increasing neurogenesis

Statins and Cognitive Outcomes After TBI

Human studies: Some human studies assessed the cognitive outcome, especially dementia after TBI. Khokhar et al. studied the relationship between post-TBI statin administration and the mortality and different associated morbidities, including stroke, depression, AD, and related dementias (ADRD) in a retrospective cohort study [25]. The study included 100,515 Medicare beneficiaries 65 years and older and survived a (TBI) hospitalization, 50,173 statin users, and 50,342 non-statin users. The authors concluded that any statin use was associated with decreased mortality (p<0.05). Any statin use was also associated with a decrease in Alzheimer's disease and related dementias (RR, 0.77; 95% CI, 0.73-0.81), and stroke (RR, 0.86; 95% confidence intervals (CI), 0.81-0.91), and depression (RR, 0.85; 95% CI, 0.79-0.90). Possible administration of other drugs during hospitalization, continuity of the statins, and lack of generalizability are limitations that faced this study.

Redelmeier et al. conducted a population-based double cohort study [26]. The study included 28,815 patients (median age = 76 years; 61.3% female) diagnosed with a concussion by the assessing physician. They excluded patients with severe TBI that required admission within two days of the head injury and patients with a history of dementia or delirium in the prior five years, 7058 (24.5%) patients received a statin; while 21,757 (75.5%) did not receive a statin. It was found that 4727 patients subsequently were diagnosed with dementia over a mean follow-up of 3.9 years, an incidence of one case per six patients. This showed a 13% reduced risk of dementia in patients who received a statin (relative risk, 0.87; 95% CI, 0.81-0.93; p<0.001). However, lack of generalizability was a limitation to this study as it included only older adults.

Li et al. published a retrospective cohort study of the association between the use of angiotensin-converting enzyme inhibitor (ACEI), simvastatin, beta-blockers, metformin, and the combinations of these drugs selected and the occurrence of probable AD after TBI [27]. The study included 733,920 patients, 15.450 patients with a history of TBI, and 718,470 non-TBI patients, TBI patients were followed for up to 18.5 years. TBI was associated with dementia and possibly AD with an odds ratio (OR) of TBI initial occurrence of 1.25 (OR 1.25 [95% CI], [1.134-1.378]) and was statistically significant (p<0.05). ACEI + statins exhibited a significantly lower risk, with hazard ratio (HR 0.35 [95%CI] [0.15-0.82]) (p<0.02) compared to statin + metformin cohort, and (HR 0.44 [95%CI] [0.15-0.82]) (p<0.01) in comparison to no treatment. Combining ACEI with statins and the small sample size of the group were the limitations of this study. A summary of the human studies showing the effect of statins on the outcomes after TBI is in Table 5.

**Table 5:** Summary of the selected human studies showing the effect of statins on the cognitive outcomes after TBI. AD: Alzheimer’s disease; TBI: Traumatic brain injury; RR: Relative risk; HR: Hazard ratio; CI: Confidence interval

Study author & year	Type & sample size	Study characteristics	Outcome & Conclusion
Khokhar et al. 2018 [25]	Cohort, 100,515	n=50,173 (statin users) , 50,342 (non-statin users), age ≥ 65	Statins ↓↓ mortality (p<0.05), AD and related dementias (RR, 0.77; 95% CI, 0.73-0.81), strokes (RR, 0.86; 95% CI, 0.81-0.91), and depression (RR, 0.85; 95% CI, 0.79-0.90) after TBI, Statins improve the outcomes and prevent secondary injury after TBI.
Redelmeier et al. 2019 [26]	Cohort, 28,815	n=28,815 concussion pts, median age=76 years; 61.3% females, n=7,058 (24.5%) statin, n=21,757 (75.5%) no statin	Statins ↓↓ risk of dementia after concussions by 6.13% (relative risk, 0.87; 95% CI, 0.81-0.93; p<0.001), Statins reduce the risk of dementia after concussions.
Li et al. 2020 [27]	Cohort, 733,920	n=733,920; n=15,450 TBI, n=718,470 non-TBI age=50-89, f/u = 18.5 years	TBI was associated with dementia & AD (OR 1.25 [95% CI], [1.134–1.378]) (p <0.05), ACEI + statins ↓↓ the risk of dementia: (HR 0.35 [95%CI] [0.15–0.82]) in comparison to statin +metformin, (HR 0.44 [95%CI] [0.15–0.82]) (p<0.01) in comparison to no treatment

Animal studies: Some studies in rats investigated the role of statins on the cognitive outcome after TBI. Darwish et al. published a study that examined the simvastatin and environmental enrichment potential effect after TBI [35]. Male Wistar rats (n=23) were divided into four groups; three groups received mild-to-moderate CCI and were treated with either saline (n=4), simvastatin (1mg/kg) orally (n=6), or environmental enrichment (EE) (n=6), while one group received sham surgery only (n=7); saline or simvastatin and was continued for 14 days. Randomized repeated measure experimental design and a Y-shaped maze were used to examine spontaneous object (SO) and temporal order (TO) memory at six, 24, 48, and 72 hours and seven, 14, 21, and 35 days post-surgery, then all subjects were sacrificed two hours after the last testing session 35 days post-TBI for a brain histopathology examination. Simvastatin reversed the TO memory deficits 14 days post-injury after a longer delay (60 minutes) (p=0.004), EE improved the animals’ SO recognition deficits seven days post-injury after a shorter delay (one minute) - only this difference approached statistical significance (p=0.09). However, this improvement was not sustained. This study concluded that simvastatin and EE are promising therapies for memory impairment after TBI.

A lab study by Mountney et al. selected simvastatin as the fourth drug tested by operation brain trauma therapy (OBTT) to assess its neuroprotective effect [36]. 128 adult male Sprague-Dawley rats were divided into sham surgery group or induced TBI group within three different models; moderate fluid percussion injury (FPI) model (n=38), CCI model (n=38), and penetrating ballistic-like brain injury (PBBI) model (n=52). TBI group in each model received either vehicle or simvastatin (1 or 5mg/kg) that was delivered orally for 14 days. In the FPI group, a hidden platform task was used to assess cognitive functions over four days (days 13 to 16 after TBI), followed by a probe trial and subsequent working memory test. The TBI groups treated with simvastatin (1mg/kg) were significantly different from sham and vehicle-treated animals at day three. It resulted in full negative points (-2.0) for this outcome in the OBTT scoring matrix, rats treated with 5mg/kg simvastatin showed improved performance. However, not significant, memory path length showed similar results. In the CCI group with the hidden platform Morris water maze (MWM) task, all TBI groups performed significantly worse than shams, and simvastatin groups showed no difference from the vehicle group. PBBI group results of the probe trial did not significantly differ between groups (p>0.05). This study shows no significant therapeutic benefit of simvastatin on any MWM parameters and cognitive outcomes after TBI, but it was worsened in the FPI group.

However, another study conducted by Mountney et al. evaluated the therapeutic efficacy of intravenous simvastatin using a varied dosing schedule on cognitive and motor function after TBI [37]. They performed PBBI on male Sprague-Dawley rats (270 to 320g; Charles River Laboratories, Raleigh, VA) (n = 8-10/group) were randomly divided into three groups; sham, vehicle, and simvastatin for four days (SV_4d) or ten days (SV_10d). Simvastatin was administered at 0.1mg/kg at 30 mins and six hours post-injury and continued once through four or 10 days post-PBBI. They used the Rotemex-5 rotarod apparatus to measure motor coordination and balance. Two weeks post-PBBI, cognitive function was assessed in a spatial learning paradigm using the MWM task. They drew blood intermittently to measure Glial fibrillary acidic protein (GFAP) and cytokines at one, four, and 24 hours post-injury. Neuro-score measures detected no differences between vehicle and simvastatin groups at any time-point (p>0.05). Also, there were no motor outcome effects following treatment with simvastatin. Initially, all injury groups showed learning deficits, though animals treated with simvastatin for a longer duration showed improved MWM performance (p<0.05). Animals that received four-day simvastatin treatment showed an intermediate effect. Moreover, the simvastatin group showed significantly reduced GFAP levels at one hour (p<0.05). It delayed GFAP systemic elevation that was sustained through 72 hours, in addition to a reduction in the systemic cytokines like interleukin-1 family (IL-1α) and interleukin-17 (IL-17) in serum such that levels were indistinguishable from sham. Simvastatin effects were sustained through 24 hours. This study provided strong evidence that IV simvastatin significantly improves memory retention, reduces neuroinflammation and the levels of circulating injury-induced GFAP. In Table 6, we summarize the results of the selected animal showing the cognitive outcomes of using statins after TBI.

**Table 6:** Summary of the selected animal trials showing the effect of statins on the cognitive outcomes after TBI. CCI: Controlled cortical impact; EE: Environmental enrichment; TBI: Traumatic brain injury; TO: Temporal order; OBTT: Operation brain trauma therapy; FPI: Fluid percussion injury; PBBI: Penetrating ballistic-like brain injury; SV: Simvastatin; MWM: Morris water maze; GFAP: Glial fibrillary acidic protein; Simvas: Simvastatin

Study author & year	Type & characteristics	Intervention	Outcome & Conclusion
Darwish et al. 2014 [35]	Animal clinical trial, male Wistar rats (n=23)	CCI + saline (n=4), CCI + simvastatin (1mg/kg) orally (n=6) CCI + EE (n=6), sham (n=7)	Simvastatin reversed TO at 14 days after (60 minutes) (p=0.004). Simvastatin is a promising therapy for memory impairment after TBI
Mountney et al. 2016 [36]	Animal clinical trial, adult male Sprague-Dawley rats (n=128)	(FPI) model (n=38) 10 sham, nine FPI + VEH, nine FP + simvas 1mg/kg, nine FPI + simvas 5mg/kg, (CCI) model (n=38) 10 sham, eight CCI + VEH, eight CCI + simvas 1mg/kg 10 CCI + simvas 5mg/kg, (PBBI) model (n=52) 14 sham, 13 PBBI + VEH 12 PBBI + simvas 1mg/kg ,13 PBBI + simvas 5mg/kg	FPI: (-2.0) for cognitive outcome in the OBTT scoring matrix CCI: simvastatin groups showed no difference from vehicle PBBI: no significant difference between any groups (p>0.05) Simvastatin showed no significant therapeutic benefit on any MWM parameters and cognitive outcomes after TBI
Mountney et al. 2016 [37]	Animal clinical trial, male Sprague-Dawley rats	Sham only PBBI + vehicle, PBBI + simvastatin IV 0.1 mg/kg initiated four days (SV_4d) or 10 days (SV_10d) (n=8-10/group)	Simvastatin ↑↑ MWM performance (p<0.05) IV Simvastatin improves memory retention, reduces neuroinflammation and the levels of circulating injury-induced GFAP after TBI

Statins’ Effect on Survival After TBI

Mansi et al. published a retrospective cohort study that included 4343 patients diagnosed with TBI in 2005 and identified 172 statin users and 4171 nonusers [28]. The statin group patients were of older age, had more comorbidities than the no statin group. This study found no statistically significant benefit or harm of statins use in patients with TBI neither in the short-term TBI outcome nor the longer-term outcomes, such as neurological disorders, substance dependence or abuse, and psychiatric disorders. However, the relatively short follow-up and the small sample size were the limitations of this study.

Some studies examined the effects of statins on the TBI outcome regarding mortality and multiple morbidities, including cognitive outcomes. Gruenbaum et al. conducted a systematic review to evaluate the recent clinical studies from 2013 through 2015 that investigated the neuroprotective roles and functional outcomes of different pharmacological agents, including statins after TBI [29]; 25 studies were included in the analysis with two studies focused on the role of statins. The results suggest that statins reduce pro-inflammatory mediators and improve functional outcomes after TBI. Also, the abrupt, unintended discontinuation of statin therapy is associated with an increased risk of mortality in the elderly cohort with TBI. However, this review was limited by the availability of human studies investigating the potential effect of statins on functional outcomes after TBI. The following Table 7 summarizes the human studies included that investigated the effect of statins on the overall survival after TBI.

**Table 7:** Summary of the human studies showing the effect of statins on the overall survival after traumatic brain injury. TBI: Traumatic brain injury

Study author & year	Type & sample size	Study characteristics	Outcome & conclusion
Mansi et al 2020 [28]	Cohort, 4343	n=4343 TBI, n=172 statin-users, n=4171 nonusers	No statistically significant benefit or harm of statins after TBI
Gruenbaum et al. 2016 [29]	Systematic review, two studies on statins, 112 patients	Systematic search investigated 15 independent pharmacological therapies, 25 studies were selected, two cohort studies on statins were included	Eight of these therapies showed neuroprotective properties, statins showed a reduction in pro-inflammatory mediators and improved functional outcomes after TBI, discontinuation of statins is associated with an increased risk of mortality in the elderly with TBI

**Figure 4:**
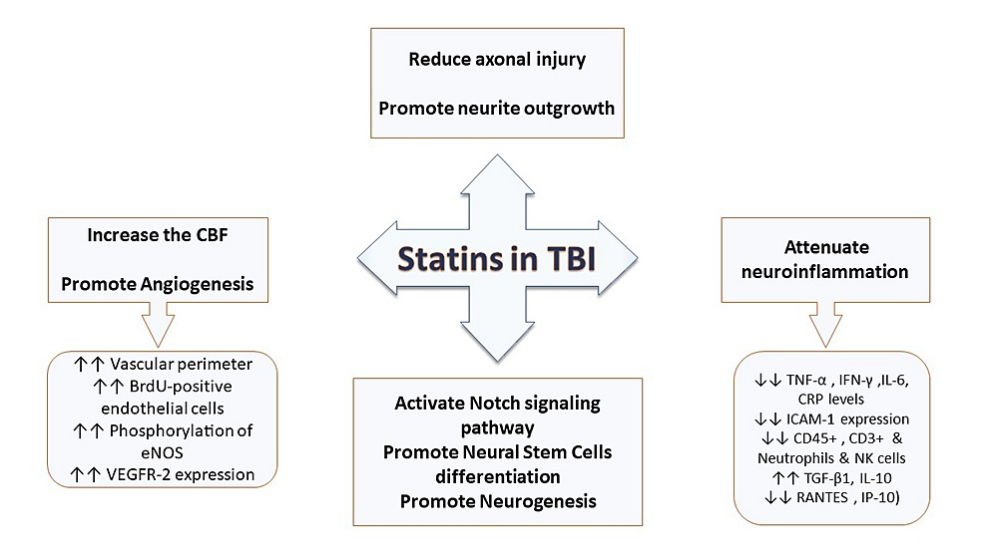
Summary of effects suggested by the selected studies of statins after TBI, including promoting neurogenesis, angiogenesis, increasing cerebral blood flow, decreasing axonal injury, and attenuating neuroinflammation. TBI: Traumatic brain injury; CBF: Cerebral blood flow; BrdU: Bromodeoxyuridine; eNOS: Endothelial nitric oxide synthase; TNF-α: Tumor necrosis factor-alpha; CRP: C-reactive protein; IFN-γ: Interferon-gamma; IL-6,10: Interleukin-6,10; NK cells: Natural killer cells; TGF-β1: Transforming growth factor-beta1; VEGFR-2: Vascular endothelial growth factor receptor-2; GFAP: Glial fibrillary acidic protein; ICAM-1: Intercellular adhesion molecule-1; RANTES: Regulated upon activation normal T-cell expressed and secreted; IP-10: Interferon-γ inducible protein

The studies included in the review showed that statins are strongly suggested to play a neuroprotective role that results in favorable overall survival, neurological and cognitive functions. This underlies multiple mechanisms summarized in Figure 4 that provide important therapeutic targets for future research and investigations.

Limitations

This study faced some limitations; first, there were limited available optimal sample-sized studies investigating the potential role of statins in humans after TBI, despite the availability of such studies in animal models. Second, most of the studies in humans were limited by lack of generalizability, sample size, short follow-up periods that could have affected the long-term outcomes; also, many subjects were lost to follow-up. Finally, no studies were conducted to compare the efficacy of different statins, dose-response, dose-duration, or different routes of administration.

## Conclusions

TBI has devastating outcomes regardless of its severity and represents a significant burden on society's long-term care. The majority of the reviewed studies showed the promising neuroprotective impact of statins and favorable results of using them in patients with TBI, particularly cognitive outcomes including Alzheimer's and non-Alzheimer's dementia. Possible therapeutic effects include its anti-inflammatory and anti-apoptotic role, promoting neurogenesis, NSC differentiation, angiogenesis, increasing the CBF, and attenuating axonal injuries in TBI patients.

Advanced studies with bigger sample sizes are needed to establish statins' safety and efficacy in treating humans with TBI. In addition, further research is needed to test the full dose-response, duration, route of administration and compare different statins to form and optimize treatment protocols.
